# Heme oxygenase-1: for better, for worse, in sickness and in health

**DOI:** 10.18632/oncotarget.4440

**Published:** 2015-06-12

**Authors:** María Emilia Solano, Petra Clara Arck

**Affiliations:** Department of Obstetrics and Fetal Medicine, Laboratory for Experimental Feto-Maternal Medicine, University Medical Center Hamburg-Eppendorf, Hamburg, Germany

Heme oxygenase (HMOX)-1, also known as heat shock protein 32, is the inducible isoform of an enzyme involved in the catabolic pathway of heme. It produces equimolar amounts of ferrous iron, carbon monoxide, and biliverdin. HMOX-1 is ubiquitously expressed and its activity has been linked to a wealth of effects, including anti-inflammation, anti-apoptosis and anti-proliferation.

Due to its anti-inflammatory, immunosuppressive properties, HMOX-1 has received considerable research attention with regard to conditions in which a tailored immune adaptation is required, e.g. upon organ transplantation, carcinogenesis or during pregnancy. In normally progressing pregnancies, the synergistic interaction between the maternal endocrine and immune responses is required to ensure a tolerogenic environment in which the semiallogenic conceptus can thrive [1]. Placental HMOX-1 is a pivotal promoter of pregnancy, e.g. via the induction of pro-angiogenic factors in the placenta [2]. In fact, reduced expression of HMOX-1 has been associated with pathological pregnancy outcomes in mammals, such as spontaneous abortion or preeclampsia.

In contrast to the beneficial effect of HMOX-1 during pregnancy, published evidence supports that HMOX-1 is over-expressed in a number of human malignancies, including renal, gastrointestinal, lung and breast cancers [3]. In the latter, HMOX-1 expression predicts a shorter overall patients’ survival [4]. However, it should be noted that the role of HMOX-1 in carcinogenesis is highly complex, likely due to the substantial heterogeneity and kinetics of the disease. Published evidence also indicates that HMOX-1 confers protection during early tumor development.

In the context of pregnancy, we have recently added evidence to the protective role of HMOX-1 by showing that placental HMOX-1 expression determines fetal growth in mice. Here, suboptimal expression led to placental insufficiency and fetal growth restriction, similar to the clinical features seen in intrauterine growth restriction in humans [5]. Functional analyses unveiled the involvement of epigenetic pathways in the reduced placental HMOX-1 expression and associated poor fetal growth, as we identified an altered DNA methylation in a cytosine-guanine dinucleotide (CpG) island around the transcription start site of the HMOX-1 gene. We could also identify that HMOX-1 interacts with the adaptive immune response by promoting the generation of a T cell subset with immunosuppressive functions, identified as CD8+CD122+ T cells. Adoptive transfer of this cell subset in partially deficient HMOX-1 mice ameliorated fetal growth restriction and promoted placental vascularization.

Strikingly, in patients with malignant melanoma, renal cell carcinoma and breast cancer, an immunosuppressive CD8+ T cell subset has also been detected in peripheral blood and among tumor-infiltrating lymphocytes [6]. These CD8+ T cells were HMOX-1– specific and superior in their immunosuppressive effects compared to e.g. conventional CD4+ regulatory T cells. Similarly, increased numbers of a regulatory CD8+ T cells subset, identified as CD28neg, are indicative for a poor survival prognosis in breast cancer patients [7].

**Figure 1:**
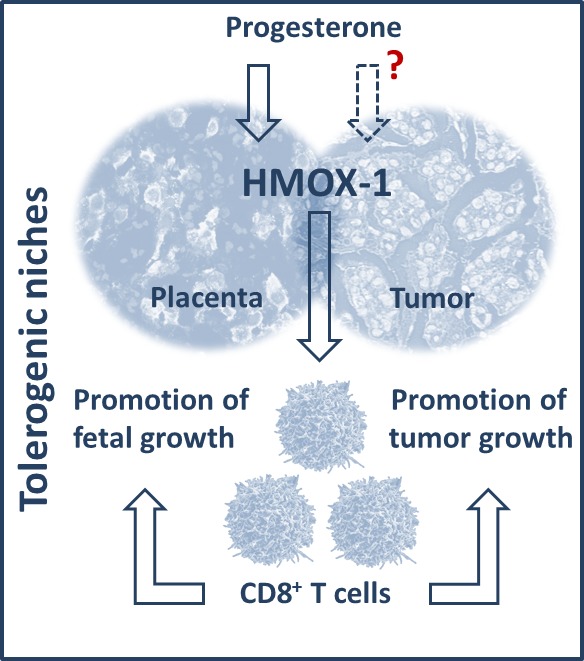
The role of HMOX-1 in creating tolerogenic niches in sickness (tumorigenesis) and health (normally progressing pregnancies).

Taken together, these findings arising from reproductive biology and oncology research endeavors strongly support that HMOX-1 is actively involved in creating a tolerogenic niche by protecting tissues from attacks of the host's immune response via the generation of immunosuppressive CD8+ T cells (Figure 1). This is clearly ‘for better’ in the context of maintaining a healthy pregnancy, but ‘for worse’ in the context of tumor growth.

Considering this increasingly recognized and critical role of HMOX-1, it is of great interest to understand pathways involved in modulating HMOX-1 tissue expression, as this may provide targets for therapeutic interventions aiming to prevent pregnancy complications or to reduce tumor progression. Under pathophysiological conditions, it has been well described that HMOX-1 can be induced by endogenous cellular stresses, such as presence of its substrate heme, endotoxin, cytokines, hypoxia, nitric oxide, and UV irradiation. We could show that one of the key hormones significantly increased during pregnancy, progesterone, up-regulates placental HMOX-1 expression in mice. Along this line, whilst it is well known that pregnancy reduces the maternal risk of breast cancer in the long term, an increased breast cancer risk during pregnancy and postpartum has been observed. This advocates the possible involvement of endogenous progesterone in the development of hormone-responsive tumor cells, e.g. in the breast, and it would now be important to assess if HMOX-1 expression is modulated by pregnancy-related hormone receptors in these malignancies.

In conclusion, HMOX-1 is pivotal modulator in a number of settings and recent insights add convincing evidence for the HMOX-1-dependent creation of tolerogenic niches (Figure 1). This unveils exiting new research avenues and strongly encourages that scientists should investigate the functional role of common-denominator markers like HMOX-1 by taking advantage of insights available from interdisciplinary approaches and perspectives.
